# Health systems integration: competing or shared mental models?

**DOI:** 10.5334/ijic.1578

**Published:** 2014-10-09

**Authors:** Jenna M. Evans

**Affiliations:** 2013. Institute of Health Policy, Management & Evaluation, University of Toronto, Canada

## Introduction

Over the past two decades, most scholars have focused on the structural and process challenges involved in delivering integrated care. The resulting knowledge on barriers and enablers has informed many positive system changes. However, researchers and practitioners continue to emphasize the seemingly intractable problems inherent in fostering collaboration and cooperation across professional and organizational boundaries. This dissertation argues that these ongoing challenges point towards the need to supplement existing frameworks and practices focused on structure, process and culture, with an understanding of the social cognitions that characterize the behaviours of actors within health care systems.

## Purpose

The aim of this dissertation was to explore the theoretical, empirical and practical utility of shared mental model (SMM) theory to the field of integrated care. SMM theory is used extensively in the team performance literature to help explain team dynamics and functioning [1]. When multiple individuals develop a common psychological structure for understanding their environment, this is referred to as an SMM [2]. SMMs allow individuals to behave in ways that are consistent and coordinated with each other in the completion of interdependent tasks [1, 2].

## Study I

The first study in this dissertation examines the evolution of integrated care strategies over 25 years [3]. Six major, interrelated shifts were identified in strategy content:

1. from a focus on horizontal integration to an emphasis on vertical integration;
2. from acute care and institution-centered models of integration to a broader focus on community-based health and social services;
3. from economic arguments for integration to an emphasis on improving quality of care and creating value;
4. from evaluations of integration using an organizational perspective to an emerging interest in patient-centered measures;
5. from a focus on modifying organizational and environmental structures to an emphasis on changing ways of working and influencing underlying cultural attitudes and norms; and
6. from integration for all patients within defined regions to a strategic focus on integrating care for specific populations.

These shifts highlight the importance of attention to meanings and perceptions (i.e., mental models) and can be used as a descriptive framework against which to assess, compare and track integrated care strategies over time.

## Study II

The second study draws from SMM theory, and an exploratory, theory-driven literature review, to explain the value of a SMMs perspective to integrated care efforts and to develop a theoretical framework of antecedents, outcomes and moderators of mental models of integrated care (MMIC) [4]. MMIC are defined as cognitive structures that represent knowledge and beliefs about integrating services. The framework suggests that mental model similarity contributes to improved processes (e.g., communication and cooperation) and performance (e.g., strategy implementation and willingness to continue working together). Two key factors are identified as precursors to mental model similarity, namely stakeholder characteristics (e.g., position/sector similarity and past integration experience) and environmental factors (e.g., funding mechanisms and system commitment to integrated care).

## Study III

The final paper validates and improves the framework of MMIC content using a two-round, web-based modified Delphi process with a diverse, pan-Canadian group of integration experts, including policymakers, planners, managers, care providers, educators, researchers and patient advocates [5]. In the first round, 90 individuals responded (52% response rate), representing a wide range of professional roles and organization types from across the continuum of care. In the second round, 68 individuals responded (75.6% response rate). The quantitative and qualitative feedback from experts was used to revise the framework. The resulting “Integration Mindsets Framework” outlines important knowledge and beliefs whose convergence or divergence across stakeholder groups may influence interprofessional and interorganizational relations. The framework consists of two overarching types of mental models, a strategy mental model, which is a conceptualization of what is being integrated and how, why and for whom it is being integrated, and a relationships mental model, which is a conceptualization of the organizations, groups and individuals involved in integration and how they are connected. These two types of mental models consist of a total of 19 knowledge-based and belief-based content areas (please refer to Evans et al. [5] for the full framework).

## Summary

**Figure 1.**
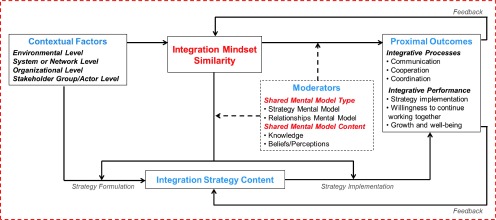
A Cognitive Perspective on Health Systems Integration: Conceptual Overview.

Figure 1 summarizes the conceptual thinking that emerged from the three studies. The construct of “Integration Mindsets” is at the heart of Figure 1 and is defined as an individual's way of thinking about integration that is based on knowledge and beliefs regarding the strategy for achieving integration (i.e., Strategy Mental Model) and the roles and relationships of those involved in the integration process (i.e., Relationships Mental Model) [5]. The similarity of Integration Mindsets is shaped by contextual factors at multiple levels [4]. The similarity of Integration Mindsets, in turn, influences strategic processes and proximal outcomes. The relationships among Integration Mindset similarity, strategic processes, and proximal outcomes are moderated by mental model type (strategy and/or relationships) and mental model content (knowledge and/or beliefs). Both formal and informal feedback from integrative processes and performance continually shape integration strategy content (i.e., what is decided) and the similarity of Integration Mindsets. Thus, shared Integration Mindsets are not a static “state”, but rather a dynamic process in which knowledge and beliefs are continuously modified and negotiated.

## Implications for integrated care

A socio-cognitive perspective on integrated care focuses attention on the evolution and interplay of meanings, interpretations, and knowledge about integration – and their potential impact on practice. Together, the studies provide theory-based, expert-validated constructs and frameworks that enable researchers and practitioners to understand, track and manage integration mindsets over time and across professional and organizational boundaries. The Integration Mindsets Framework can be used to direct and focus early discussions and planning efforts among team members or partnering organizations and to assess organizational or system readiness for integration. Awareness of the extent to which integration mindsets are shared and where similarities and differences lie can also help guide change management interventions. Finally, shared integration mindsets may be used as one indicator, among many, of a successful and sustainable integration activity.

The results presented in this review are based on the author’s thesis presented at the University of Toronto on 5 December 2013.
